# Alterations in Gastric Mucosal Microbiota in Gastric Carcinogenesis: A Systematic Review and Meta-Analysis

**DOI:** 10.3389/fmed.2021.754959

**Published:** 2021-12-03

**Authors:** Yingyun Yang, Ruoyu Ji, Xinyu Zhao, Xinyuan Cao, Qiang Wang, Qingwei Jiang, Yizhen Zhang, Weiyang Zheng, Xi Wu, Aiming Yang

**Affiliations:** ^1^Department of Gastroenterology, Peking Union Medical College Hospital (PUMCH), Chinese Academy of Medical Sciences & Peking Union Medical College (CAMS & PUMC), Beijing, China; ^2^National Clinical Research Center for Digestive Diseases, Department of Clinical Epidemiology and Evidence-based Medicine, Beijing Friendship Hospital, Capital Medical University, Beijing, China

**Keywords:** gastric microbiota, gastric cancer, dysbiosis, stomach microhabitat, meta-analysis

## Abstract

**Background:** The gastric microbiota profile alters during gastric carcinogenesis. We aimed to identify the alterations in the alpha diversity and relative abundance of bacterial phyla and genera of gastric microbiota in the development of gastric cancer (GC).

**Methods:** The systematic review was performed based on a published protocol with the registration number CRD42020206973. We searched through PubMed, EMBASE and Cochrane databases, as well as conference proceedings and references of review articles (May 2021) for observational studies reporting either the relative abundance of bacterial phyla or genera, or alpha diversity indexes in both GC and non-cancer groups. Selection of studies and data extraction were performed independently by two researchers, with disagreements resolved through discussion. Risk of bias was assessed using the self-modified Newcastle-Ottawa Scale. Results of random-effects meta-analyses were presented as mean differences (MD).

**Results:** Our systematic review included 751 GC patients and 792 non-cancer patients from 14 case-control studies. Gastric cancer group had fewer operational taxonomic units (OTUs) (MD = −68.52, 95%CI: −126.65 to −10.39) and a lower Simpson index (MD = −0.13, 95%CI: −0.20 to −0.07) compared with non-cancer group. At the phylum level, gastric cancer group had a higher abundance of *Firmicutes* (MD = 7.11, 95%CI: 1.76 to 12.46). At the genus level, *Streptococcus* (MD = 3.03, 95%CI: 0.07 to 6.00) and *Lactobacillus* (MD = 5.15, 95%CI: 1.27 to 9.04) were found to be enriched in GCgroup. The relative abundance of the rest bacterial phyla or genera analyzed in our study did not significantly differ between two groups. Subgroup analyses indicated that the source of samples was the major source of interstudy heterogeneity.

**Conclusion:** This systematic review suggested that gastric microbiota dysbiosis occurred in gastric carcinogenesis, with alpha diversity declined and microbiota composition altered.

## Highlights

Increasing evidence has illustrated that the diversity and composition of gastric mucosal microbiota alter in gastric carcinogenesis. However, the changing pattern remains poorly understood as the findings differed across studies. This systematic review was performed based on a peer-reviewed and published protocol with the aim of evaluating the differences in the alpha diversity and relative abundance of bacterial phyla and genera between GC tissues and non-cancer tissues. Based on 14 studies with a total of 1,543 patients, our results indicated that alpha diversity declined in GC tissues and the microbiota composition altered at both phylum and genus levels. Our findings provided new insights into the involvements of gastric microbiota in gastric carcinogenesis as well as the prevention, diagnosis and treatment strategies for GC at the microbiological level.

## Introduction

The human gastrointestinal tract is a complicated ecosystem harboring numerous microorganisms. Gut microbes play essential roles in diverse physiological processes and are also involved in disease occurrence and development (1). The stomach had long been considered as a sterile organ until the discovery of *Helicobacter pylori (H. pylori)*. With the advancement of high-throughput sequencing technology, a unique and complex gastric microbiota composition has been gradually uncovered (2).

Gastric cancer (GC) is the fifth most prevalent and the third most lethal malignancy worldwide (3). As postulated by Correa's model, normal gastric mucosa goes through the progressive stages from non-atrophic gastritis, atrophic gastritis, intestinal metaplasia, intraepithelial neoplasia and eventually to GC (4). Numerous studies have implicated *H. pylori* infection in this multistep process (5). However, only a minor fraction of patients with *H. pylori* infection ultimately develop GC (6), and the eradication of *H. pylori* does not completely prevent carcinogenesis (7, 8). Therefore, a growing number of studies have focused on the contribution of gastric microbiota dysbiosis to GC development (9, 10).

To date, the knowledge on the role of gastric microbiota dysbiosis in gastric tumorigenesis is still insufficient. Studies have noticed consecutive shifts in gastric microbiota profile during cancer development, with microbial diversity and composition changed (9, 11). However, the gastric microbiota is diverse and dynamic, which could be affected by multiple factors and differs geographically and ethnically (12, 13). Discrepancies across studies and limited sample sizes have compromised a clear understanding of this issue. Moreover, identifying the changes in gastric microbiota profile may help in prevention, diagnosis and treatment of GC. This underscores the need to perform a systematic review and meta-analysis for quantitative evaluation of changes in the diversity and composition of gastric mucosal microbiota in gastric carcinogenesis.

## Materials and Methods

We performed the systematic review based on a peer-reviewed and published protocol (14) with the registration number CRD42020206973 and complied with the Preferred Reporting Items for Systematic Review and Meta-Analysis (PRISMA) statement (15). Reporting items were detailed in the PRISMA checklist (Supplementary Material).

The purpose of this review was to evaluate the differences in the diversity of gastric microbiota and relative abundance of bacterial phyla and genera between GC tissues and non-cancer tissues.

### Literature Search

We searched through PubMed, EMBASE and Cochrane databases. The search strategy in PubMed was: ((“microbiome” OR “microbial” OR “microbiota” [MeSH Terms]) OR “microflora” OR “bacterial” OR “dysbiosis”) AND (“gastric” [MeSH Terms] OR “stomach” OR “upper digestive tract” OR “upper gastrointestinal tract”) AND ((“lesion” OR “cancer” [MeSH Terms] OR “neoplasia” OR “neoplasms” OR “malignancy” OR “tumor” OR “carcinoma” OR “adenocarcinoma” OR “premalignancy” OR “premalignant” OR “tumorigenesis” OR “carcinogenesis”) OR “intestinal metaplasia” OR “gastritis”). The search strategy was adapted for EMBASE and Cochrane databases. We also searched conference proceedings and the references of review articles for relevant studies. The last search update was May 2021.

### Selection of Studies

We included observational human studies. Eligible studies must contain both GC tissues and non-cancer tissues that were confirmed by clinical and histological evaluations. Histological diagnoses of non-cancer tissues complied with the updated Sydney system (16) and the revised Vienna classification system (17). The source of samples was limited to gastric biopsy samples (surgical or endoscopic). The sequencing technology was limited to high-throughput sequencing methods. Regarding the phenomenon of interest, studies must report either the relative abundance of bacteria at the phylum or genus level, or at least one of the alpha diversity indexes (the number of operational taxonomic units (OTUs), Shannon index, Chao 1, Simpson index, etc.) in both groups. Detailed inclusion and exclusion criteria were described in the published study protocol (14). Study selection was conducted by two researchers (YYY and RYJ) independently, with disagreements resolved through discussion.

### Data Extraction

We extracted study information including publication (authors, year, journal title), study design (patient inclusion and exclusion criteria, source of samples, grouping and the sample size of each, sequencing technology) and bias control. We extracted patient characteristics including demographics (age, sex, country or region, race/ethnicity, comorbidities), lesion location, clinical and histological diagnosis and *H. pylori* infection status (determined by ^13^C urea breath test or histological assessment). We also extracted outcome data including relative abundance of bacterial phyla or genera and alpha diversity indexes.

We made full use of available materials for data extraction. If required information was not clearly or completely recorded, we contacted the corresponding author and co-authors via e-mail. Data extraction was conducted by two researchers (YYY and RYJ) independently, with disagreements resolved through discussion.

### Risk of Bias Assessment

We assessed the risk of bias using a self-modified Newcastle-Ottawa Scale (NOS) (14) which was adapted with the intention of best evaluating our phenomenon of interest (Supplementary Material). The risk of bias was evaluated from three domains: selection, comparability and exposure (or outcome), and each study was awarded with a maximum of 11 scores. Risk of bias assessment was conducted by two researchers (YYY and RYJ) independently, with disagreements resolved through discussion.

### Statistical Analysis

Basic characteristics and the phenomenon of interest of included studies were firstly tabulated. The mean differences [MD] with 95% confidence intervals [CI] were calculated as our effect measurements. If data were reported as the median with interquartile range, we converted them into the mean with standard deviation through a recommended formula (18). Considering large interstudy heterogeneity, we utilized the random-effects model. We evaluated heterogeneity across studies using the Cochrane chi-square (χ2) and quantified with the I^2^ statistics (19). I^2^ values of 25, 50 and 75% represented low, moderate and high heterogeneity, respectively (20). Publication bias was statistically examined by Egger's test (21). We conducted the following subgroup analyses to explore potential sources of heterogeneity: mean age, *H. pylori* infection status, study population, source of samples, sample size and study quality. Univariate meta-regression analyses were furthermore conducted to identify heterogeneity sources across studies. Multivariate meta-regression analyses were not performed due to limited number of included studies. All analyses except Egger's test and meta-regression analyses were performed using Review Manager 5.3.3 (Nordic Cochrane Centre, Copenhagen, Denmark) and STATA version 16 (StataCorp, College Station, TX) was used for Egger's test and meta-regression analyses. *P* < 0.05 was considered statistically significant.

## Results

**Figure 1:**
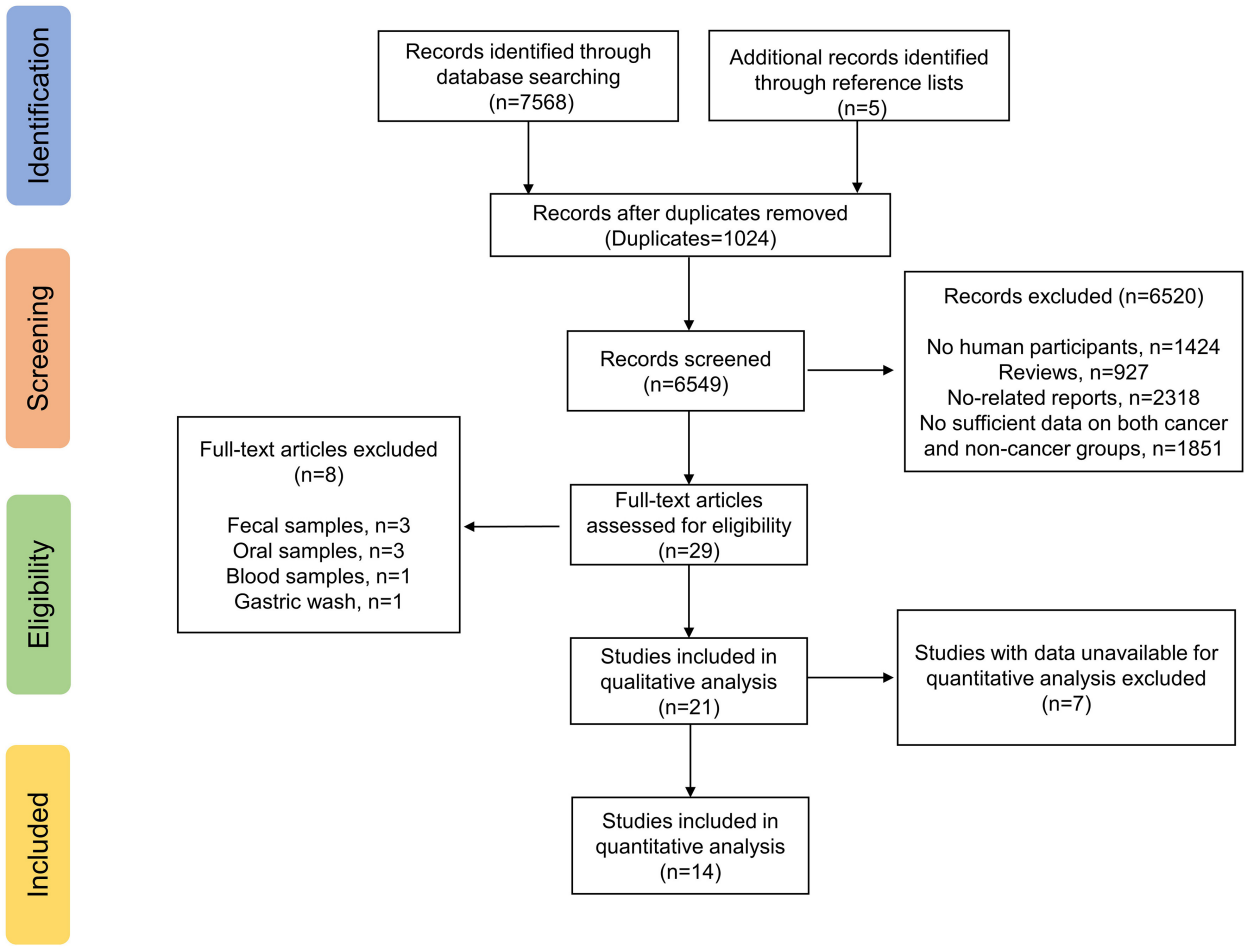
Study flow chart.

The electronic search yielded a total of 7,568 potentially relevant studies, and 5 additional studies were identified through reference lists (Figure 1). All records were imported into Endnote with 1,024 duplicates removed. After evaluating the eligibility of the studies by reading the titles and abstracts, 6,520 studies were eliminated. Studies evaluating fecal samples (8, 22, 23), oral samples (24–26), blood samples (27) or gastric wash samples (28) were further excluded. Among the remaining 21 studies, 14 reported sufficient data available for meta-analysis (29–42), while seven did not (43–49). We have so far contacted the corresponding authors and other co-authors for additional information three times, unfortunately we received no reply. Therefore, 14 studies met our inclusion criteria and were ultimately included.

All included studies were case-control studies with a median NOS score of 8 (Range: 5–9) (Table 1). Altogether, 1,543 patients, with 751 GC patients and 792 non-GC patients were included. Only one study enrolled a mixture of Chinese and Mexican population (37), while patients of the remaining studies were composed of pure Asian populations. None of the included studies enrolled patients who had recently received antibiotic treatments or chemotherapy prior to recruiments. The samples in the reviewed studies were obtained either as endoscopic biopsies (29–34, 36, 39–42) or surgical biopsies (35, 37, 38). High-throughput sequencing methods were applied by all studies.

**Table 1:** Basic characteristics of included studies.

**References**	**Non-GC Controls**					**GC (n)**	**Country/Region**	**Female proportion** **(%)**	**Mean age** **(years)**	**Adjusted variables**	**Prior antibiotics use**	**Prior chemotherapy**	**Samples**	**Sequencing methods**	**^**#**^NOS**
	**HC (n)**	**NAG (n)**	**AG (n)**	**IM (n)**	**Others (n)**										
Castaño-Rodríguez et al. (29)	-	-	-	-	Functional dyspepsia (20)	12	Malaysia and Singapore	53.1	54.3	-	Not within 2 months	-	Endoscopic biopsy	16S rRNA gene sequencing	8
Gantuya et al. (30)	20	20	40	40	-	48	Mongolia	30.0	46.4	-	Not within 1 months	-	Endoscopic biopsy	16S rRNA gene sequencing	9
Hsieh et al. (31)	-	9	-	7	-	11	Taiwan	55.6	50.7	-	-	-	Endoscopic biopsy	16S rDNA gene sequencing	5
Jo et al. (32)	-	-	-	-	Not detailed (29)	34	South Korea	42.9	58.6	-	Not within 3 months	-	Endoscopic biopsy	454 Pyrosequencing	8
Li et al. (33)	8	9	-	18	-	14	Hong Kong	67.3	49.1	Age	Not within 1 months	-	Endoscopic biopsy	16S rDNA gene sequencing	8
Park et al. (34)	-	62	-	21	-	55	South Korea	55.2	41.0	-	Not within 3 months	-	Endoscopic biopsy	16S rRNA gene sequencing	7
Tseng et al. (35)	-	-	-	-	Peritumor tissues (6)	6	Taiwan	-	-	-	Not within 1 months	No	Surgical biopsy	16S rRNA gene sequencing	6
Wang et al. (36)	-	6	-	-	-	6	China	39.7	55.8	-	Not within 1 months	-	Endoscopic biopsy	454 Pyrosequencing	8
Yu et al. (37)	-	-	-	-	Peritumor tissues (131)	131	China and Mexico	31.8	62.7	Individual matching	-	No	Surgical biopsy	16S rRNA gene sequencing	7
Chen et al. (38)	-	-	-	-	Peritumor tissues (62)	62	China	25.8	60.0	-	Not within 1 months	No	Surgical biopsy	16S rRNA gene sequencing	9
Gunathilake et al. (39)	-	-	-	-	Not detailed (288)	268	South Korea	36.5	52.6	Age, smoking, first-degree family history of GC, regular exercise, education, occupation, monthly income, and total energy intake.	-	-	Endoscopic biopsy	16S rRNA gene sequencing	9
Wang et al. (40)	30	21	-	27	Intraepithelial neoplasia (25)	29	China	42.4	54.2	-	Not within 1 months	No	Endoscopic biopsy	16S rRNA gene sequencing	8
Wang et al. (41)	-	60	-	-	-	60	China	29.2	55.9	-	Not within 2 months	-	Endoscopic biopsy	16S rRNA gene sequencing	7
Zhang et al. (42)	-	17	10	-	Intraepithelial neoplasia (5)	15	China	42.6	63.0	-	-	-	Endoscopic biopsy	16S rRNA gene sequencing	7

*GC, gastric cancer; HC, health control; NAG, non-atrophic gastritis; AG, atrophic gastritis; IM, intestinal metaplasia*.

# *Scored by a self-modified Newcastle-Ottawa Scale with a maximum score of 11*.

Regarding the phenomenon of interest (Table 2), 10 studies (32–40, 42) reported at least one of the alpha diversity indexes and nine studies (29–31, 34, 37, 39–42) reported the relative abundace of bacteria at the phylum or genus level. Four alpha diversity indexes (OTUs, Chao 1, Shannon index, Simpon index), five bacterial phyla (*Proteobacteria, Firmicutes, Bacteroidetes, Actinobacteria, Fusobacteria*) and eight bacterial genera (*Helicobacter, Streptococcus, Lactobacillus, Veillonella, Prevotella, Sphingomonas, Fusobacterium, Neisseria*) were further analyzed in the quantitative analysis.

**Table 2:** Phenomenon of interest reported by included studies.

**References**	**Alpha diversity indexes**	**Bacterial phyla**	**Bacterial genera**
Castaño-Rodríguez et al. (29)	-	*Proteobacteria, Firmicutes, Bacteroidetes, Actinobacteria, Fusobacteria*	*Helicobacter, Streptococcus, Lactobacillus, Veillonella, Prevotella, Sphingomonas, Fusobacterium, Neisseria*
Gantuya et al. (30)	-	*-*	*Helicobacter, Lactobacillus*
Hsieh et al. (31)	-	*-*	*Helicobacter, Streptococcus, Lactobacillus, Veillonella, Prevotella, Sphingomonas, Fusobacterium, Neisseria*
Jo et al. (32)	OTUs, Chao 1, Shannon index, Simpson index	*-*	*-*
Li et al. (33)	OTUs, Shannon index	*-*	*-*
Park et al. (34)	OTUs, Chao 1, Shannon index, Simpson index	*Proteobacteria, Firmicutes, Bacteroidetes, Actinobacteria, Fusobacteria*	*Helicobacter, Streptococcus, Lactobacillus, Veillonella, Prevotella, Sphingomonas, Fusobacterium, Neisseria*
Tseng et al. (35)	OTUs, Chao 1, Shannon index, Simpson index	*-*	*-*
Wang et al. (36)	Chao 1, Shannon index	*-*	*-*
Yu et al. (37)	OTUs, Shannon index	*Proteobacteria, Firmicutes, Bacteroidetes, Fusobacteria*	*Helicobacter, Streptococcus, Fusobacterium*
Chen et al. (38)	Chao 1, Shannon index	*-*	*-*
Gunathilake et al. (39)	Shannon index	*-*	*Helicobacter, Prevotella*
Wang et al. (40)	OTUs, Chao 1, Shannon index, Simpson index	*Proteobacteria, Firmicutes, Bacteroidetes, Actinobacteria, Fusobacteria*	*-*
Wang et al. (41)	-	*-*	*Sphingomonas*
Zhang et al. (42)	OTUs	*Proteobacteria, Firmicutes, Bacteroidetes, Actinobacteria, Fusobacteria*	*Helicobacter, Streptococcus, Lactobacillus, Veillonella, Prevotella, Sphingomonas, Fusobacterium, Neisseria*

*OTUs, operational taxonomic units*.

### Alpha Diversity Indexes

**Figure 2:**
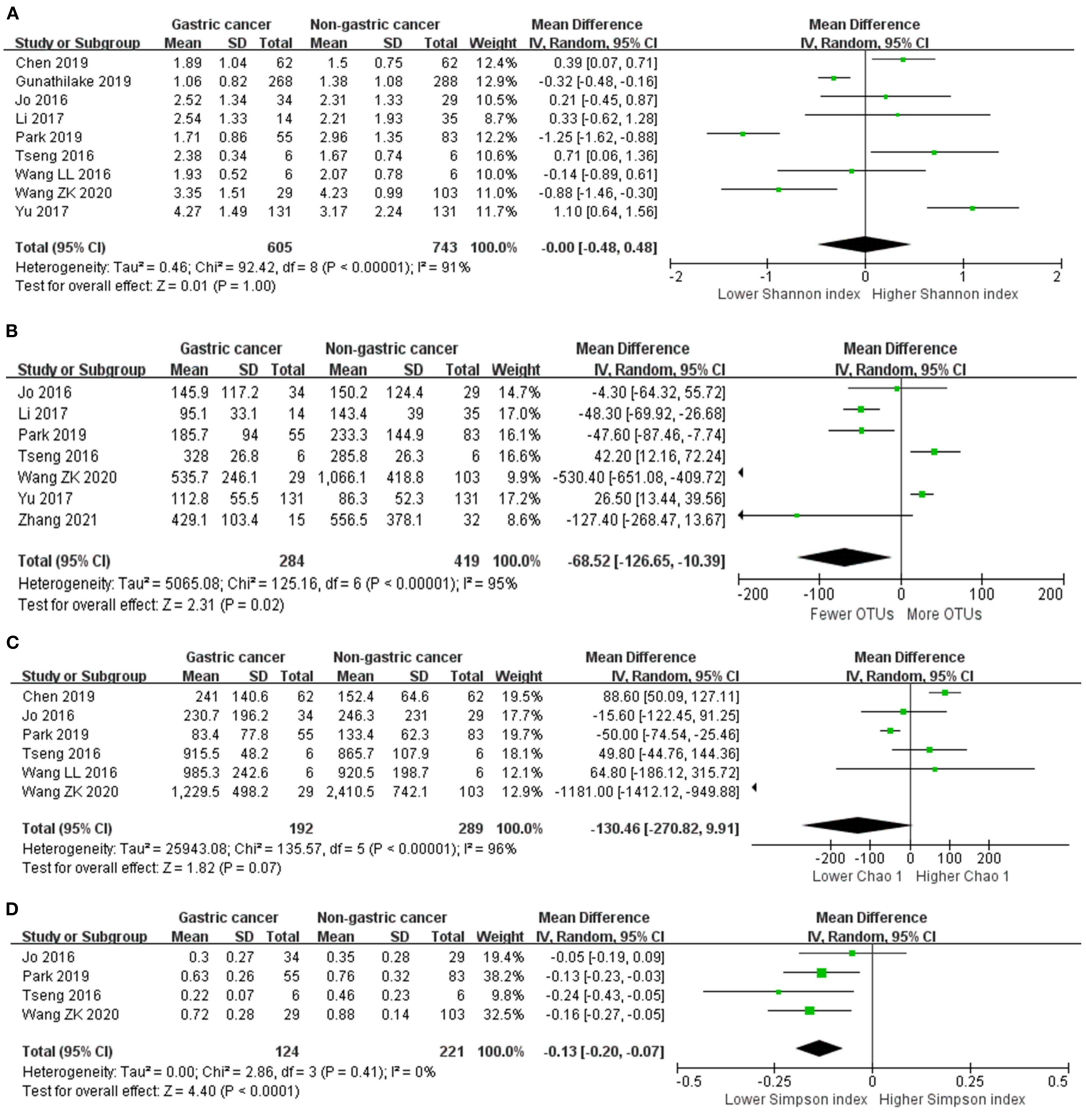
Forest plot for changes in alpha diversity indexes including Shannon index **(A)**, OTUs **(B)**, Chao 1 **(C)**, and Simpson index **(D)** between gastric cancer and non-gastric cancer groups.

We performed random-effects meta-analyses based on nine studies (32–40) evaluating Shannon index, six studies (32, 34–36, 38, 40) evaluating Chao 1, seven studies (32–35, 37, 40, 42) evaluating OTUs and four studies (32, 34, 35, 40) evaluating Simpson index (Figure 2). The microbiome of GC group had similar Shannon index compared with non-cancer group (MD = 0.00, 95%CI: −0.48 to 0.48, *P* > 0.99, I^2^ = 91%). GC group had siginificantly fewer OTUs compared with non-cancer group (MD = −68.52, 95%CI: −126.65 to −10.39, *P* = 0.02; I^2^ = 95%). The decrease of Chao 1 in GC group was not siginificant (MD = −130.46, 95%CI: −270.82 to 9.91, *P* = 0.07; I^2^ = 96%). Simpson index siginificantly declined in the GC group (MD = −0.13, 95%CI: −0.20 to −0.07, *P* < 0.001), with no evidence of between study heterogeneity (I^2^ = 0%).

Egger's test suggested no significant publication bias for Shannon index (*P* = 0.499), Chao 1 (*P* = 0.857), OTUs (*P* = 0.459) and Simpson index (*P* = 0.560).

### Bacterial Phylum

**Figure 3:**
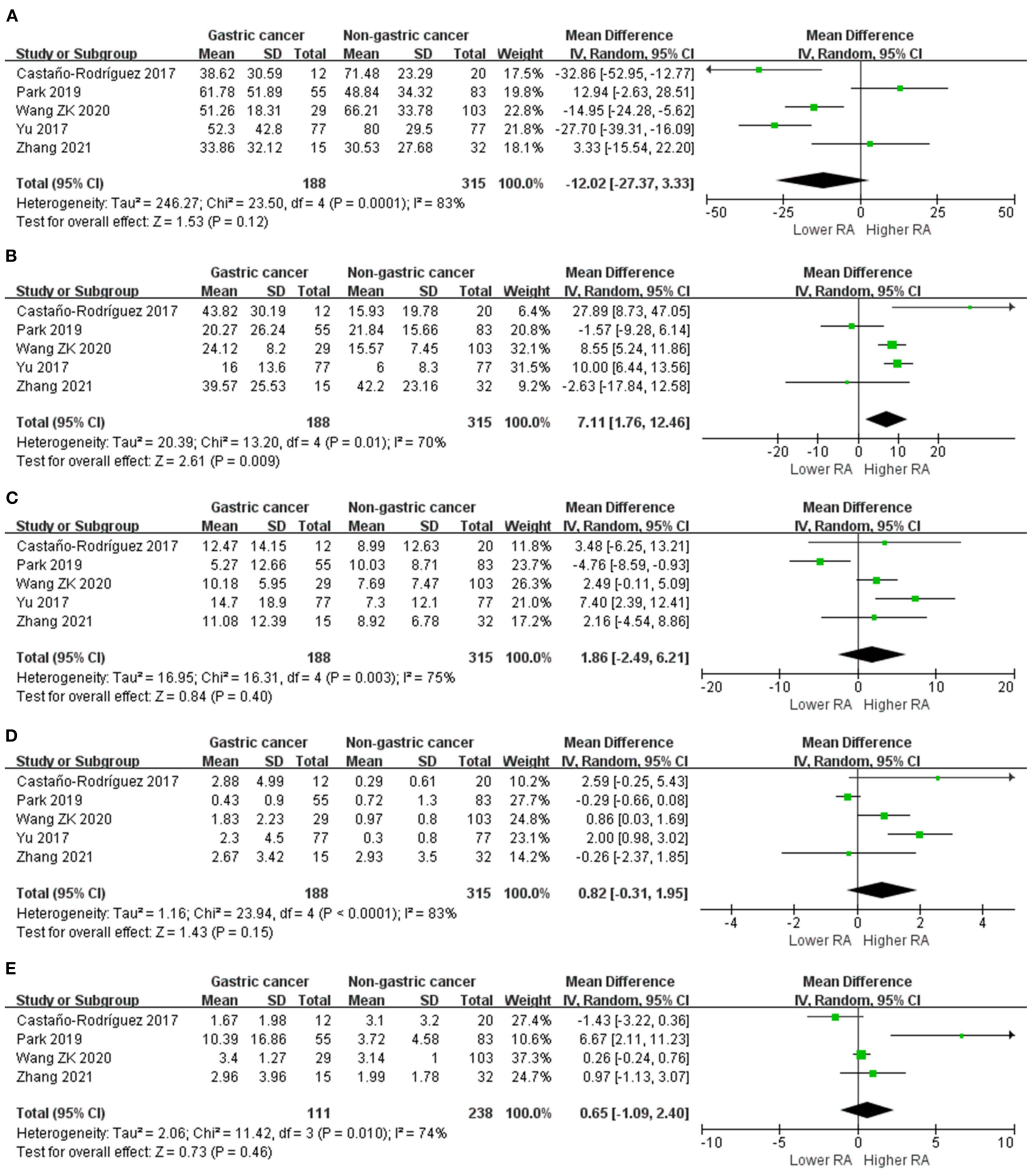
Forest plot for changes in relative abundance of bacterial phyla including *Proteobacteria* **(A)**, *Firmicutes* **(B)**, *Bacteroidetes* **(C)**, *Fusobacteria* **(D)**, and *Actinobacteria* **(E)** between gastric cancer and non-gastric cancer groups.

We performed random-effects meta-analyses based on five studies (29, 34, 37, 40, 42) evaluating the relative abundance of *Proteobacteria, Firmicutes, Bacteroidetes* and *Fusobacteria*, and four (29, 34, 40, 42) studies evaluating the relative abundance of *Actinobacteria* (Figure 3). The microbiome of GC group was composed of less *Proteobacteria* compared with non-cancer group (MD = −12.02, 95%CI: −27.37 to 3.33, *P* = 0.12; I^2^ = 83%), however, the difference was not significant. Conversely, *Firmicutes* was significantly enriched in GC group (MD = 7.11, 95%CI: 1.76 to 12.46, *P* = 0.009; I^2^ = 70%). Non-siginificant difference was found in the relative abundance of *Bacteroidetes* (MD = 1.86, 95%CI: −2.49 to 6.21, *P* = 0.40; I^2^ = 75%), *Fusobacteria* (MD = 0.82, 95%CI: −0.31 to 1.95, *P* = 0.15; I^2^ = 83%) and *Actinobacteria* (MD = 0.65, 95%CI: −1.09 to 2.40, *P* = 0.46; I^2^ = 74%) between two groups.

Egger's test suggested no significant publication bias for the relative abundance of *Proteobacteria* (*P* = 0.742), *Firmicutes* (*P* = 0.853), *Bacteroidetes* (*P* = 0.835), *Fusobacteria* (*P* = 0.823) and *Actinobacteria* (*P* = 0.197).

### Bacterial Genus

Random-effects meta-analysis based on seven studies (29–31, 34, 37, 39, 42) revealed a non-significantly lower abundance of *Helicobacter* in GC group (*MD* = −13.40, 95%CI: −28.24 to 1.45, *P* = 0.08, I^2^ = 89%). Regarding other bacterial genera, *Streptococcus* (*MD* = 3.03, 95%CI: 0.07 to 6.00, *P* = 0.04; I^2^ = 66%) and *Lactobacillus* (*MD* = 5.15, 95%CI: 1.27 to 9.04, *P* = 0.009; I^2^ = 40%) were found to be enriched in GC group. While for *Veillonella, Prevotella, Sphingomonas, Fusobacterium* and *Neisseria*, no significant differences were found between two groups (Supplementary Material).

### Subgroup Analyses and Meta-Regression Analyses

Results of subgroup analyses and univariate meta-regression analyses indicated that mean age, sources of samples, study population, sample size, study quality and *H. pylori* infection status all contributed to heterogeneities across studies to varying degrees (Table 3; Supplementary Material).

**Table 3:** Subgroup analyses and univariate meta-regression analyses of changes in alpha diversity indexes and relative abundance of bacterial phyla.

**Group**	**Subgroups**	**Studies (n)**	**^**#**^MD [95% CI]**	**I^**2**^ (%)**	**^**&**^${\text{I}}_{\text{sub}}^{\text{2}}$ (%)**	* *P*
**Shannon index**						
Age	Mean age <55 years	4	−0.60 [−1.20, 0.00]	88	0	0.44
	Mean age ≥55 years	4	0.44 [−0.04, 0.93]	71		
Sources of samples	Surgical biopsies	3	0.71 [0.24, 1.18]	68	91	<0.001
	Endoscopic biopsies	6	−0.14 [−0.67, 0.40]	87		
Study population	Asian population	9	0.05 [−0.46, 0.56]	92	0	0.66
	Non-Asian population	1	0.22 [−0.32, 0.76]	-		
Sample size	Sample size <100	4	0.30 [−0.06, 0.67]	0	37	0.21
	Sample size ≥100	5	−0.19 [−0.86, 0.48]	95		
Study quality	<8 scores by NOS	3	0.18 [−1.45, 1.80]	97	0	0.76
	≥8 scores by NOS	6	−0.09 [−0.49, 0.31]	78		
*H. pylori* infection status	Negative	4	−0.48 [−1.95, 0.99]	96	0	0.42
	Positive	4	0.16 [−0.29, 0.60]	50		
**Chao 1**						
Age	Mean age <55 years	2	−609.42 [−1717.72, 498.87]	99	28	0.24
	Mean age ≥55 years	3	56.71 [−15.79, 129.22]	38		
Sources of samples	Surgical biopsies	2	83.08 [47.41, 118.75]	0	79	0.03
	Endoscopic biopsies	4	−282.36 [−605.53, 40.82]	97		
Sample size	Sample size <100	2	21.08 [−49.74, 91.89]	0	81	0.02
	Sample size ≥100	4	−224.11 [−419.91, −28.31]	98		
Study quality	<8 scores by NOS	2	−10.98 [−106.43, 84.48]	75	33	0.22
	≥8 scores by NOS	4	−249.00 [−619.13, 121.14]	97		
*H. pylori* infection status	Negative	2	−43.32 [−56.92, −27.72]	0	88	0.004
	Positive	2	10.74 [−22.67, 44.16]	0		
**OTUs**						
Age	Mean age <55 years	3	−119.10 [−220.42, −17.79]	97	75	0.05
	Mean age ≥55 years	3	−2.43 [−57.72, 52.86]	63		
Sources of samples	Surgical biopsies	2	28.99 [17.02, 40.97]	0	94	<0.001
	Endoscopic biopsies	5	−133.17 [−228.04, −38.31]	95		
Study Population	Asian population	7	−69.62 [−132.49, −6.75]	95	80	0.02
	Non-Asian population	1	6.50 [−13.61, 26.61]	–		
Sample size	Sample size <100	4	−19.06 [−81.14, 43.03]	88	64	0.10
	Sample size ≥100	3	−163.92 [−322.46, −5.38]	98		
Study quality	<8 scores by NOS	4	−0.39 [−43.41, 42.64]	84	71	0.06
	≥8 scores by NOS	3	181.74 [−368.95, 5.47]	97		
*H. pylori* infection status	Negative	4	−39.19 [−76.69, 1.32]	76	0	0.41
	Positive	4	−14.90 [−56.73, 26.93]	69		
**Simpson index**						
Age	Mean age <55 years	2	−0.05 [−0.19, 0.99]	0	30	0.23
	Mean age ≥55 years	1	−0.14 [−0.22, −0.07]	-		
Sources of samples	Surgical biopsies	1	−0.24 [−0.43, 0.05]	-	21	0.23
	Endoscopic biopsies	3	−0.12 [−0.19, −0.06]	0		
Sample size	Sample size <100	2	−0.13 [−0.32, 0.05]	60	0	0.91
	Sample size ≥100	2	−0.14 [−0.22, −0.07]	0		
Study quality	<8 scores by NOS	2	−0.15 [−0.24, −0.07]	0	0	0.58
	≥8 scores by NOS	2	−0.11[−0.22, −0.01]	36		
*H. pylori* infection status	Negative	2	−0.14 [−0.44, 0.16]	88	0	0.39
	Positive	2	0.07 [−0.29, 0.43]	89		
**Relative abundance of** ***Proteobacteria***						
Age	Mean age <55 years	3	−11.08 [−33.64, 11.49]	86	0	0.92
	Mean age ≥55 years	2	−13.11 [−43.47, 17.24]	87		
Sources of samples	Endoscopic biopsies	4	−7.65 [−25.48, 10.17]	82	71	0.06
	Surgical biopsies	1	−27.70 [−39.31, −16.09]	-		
Sample size	Sample size <100	2	−14.59 [−50.06, 20.87]	85	0	0.85
	Sample size ≥100	3	−10.62 [−30.63, 9.39]	88		
Study quality	<8 scores by NOS	3	−4.34 [−31.40, 22.73]	90	11	0.29
	≥8 scores by NOS	2	−21.60 [−38.56, −4.64]	60		
**Relative abundance of** ***Firmicutes***						
Age	Mean age <55 years	3	8.36 [−2.27, 18.98]	80	0	0.76
	Mean age ≥55 years	2	5.94 [−5.62, 17.50]	60		
Sources of samples	Endoscopic biopsies	4	6.17 [−2.98, 15.31]	74	0	0.44
	Surgical biopsies	1	10.00 [6.44, 13.56]	-		
Sample size	Sample size <100	2	12.05 [−17.84, 41.94]	83	0	0.74
	Sample size ≥100	3	6.90 [2.06, 11.75]	72		
Study quality	<8 scores by NOS	3	3.21 [−6.35, 12.78]	78	30	0.23
	≥8 scores by NOS	2	15.82 [−2.54, 34.19]	74		
**Relative abundance of** ***Bacteroidetes***						
Age	Mean age <55 years	3	−0.08 [−5.83, 5.66]	80	47	0.17
	Mean age ≥55 years	2	5.27 [0.22, 10.31]	34		
Sources of samples	Endoscopic biopsies	4	0.34 [−4.13, 4.81]	70	76	0.04
	Surgical biopsies	1	7.40 [2.39, 12.41]	-		
Sample size	Sample size <100	2	2.58 [−2.93, 8.10]	0	0	0.81
	Sample size ≥100	3	1.57 [−4.53, 7.67]	88		
Study quality	<8 scores by NOS	3	1.45 [−6.48, 9.39]	93	0	0.80
	≥8 scores by NOS	2	2.56 [0.04, 5.07]	0		
**Relative abundance of** ***Fusobacteria***						
Age	Mean age <55 years	3	0.54 [−0.65, 1.73]	79	0	0.68
	Mean age ≥55 years	2	1.07 [−1.11, 3.25]	72		
Sources of samples	Endoscopic biopsies	4	0.38 [−0.61, 1.37]	69	80	0.03
	Surgical biopsies	1	2.00 [0.98, 3.02]	-		
Sample size	Sample size <100	2	1.00 [−1.77, 3.78]	60	0	0.89
	Sample size ≥100	3	0.79 [−0.55, 2.13]	90		
Study quality	<8 scores by NOS	3	0.53 [−1.19, 2.24]	88	0	0.46
	≥8 scores by NOS	2	1.17 [−0.13, 2.47]	24		
**Relative abundance of** ***Actinobacteria***						
Age	Mean age <55 years	3	0.79 [−1.70, 3.27]	82	0	0.91
	Mean age ≥55 years	1	0.97 [−1.13, 3.07]	-		
Sample size	Sample size <100	2	−0.29 [−2.64, 2.05]	66	0	0.33
	Sample size ≥100	2	3.05 [−3.18, 9,28]	87		
Study quality	<8 scores by NOS	2	3.44 [−2.09, 8.98]	80	40	0.20
	≥8 scores by NOS	2	−0.36 [−1.95, 1.24]	68		

# *A positive MD represents a higher relative abundance in gastric cancer group*.

& *Heterogeneity across subgroups*.

* *P value of univariate meta-regression analyses which test for subgroup differences*.

Sources of samples were the major source of heterogeneity for Shannon index and OTUs. In the surgical subgroup, GC tissues had higher Shannon index (*MD* = 0.71, 95%CI: 0.24 to 1.18) and more OTUs (*MD* = 28.99, 95%CI: 17.02 to 40.97) compared with non-cancer tissues. However, in the endoscopic subgroup, GC tissues had similar Shannon index (*MD* = −0.14, 95%CI: −0.67 to 0.40) and less OTUs (*MD* = −133.17, 95%CI: −228.04 to −38.31) compared with non-cancer tissues. The study population was also a source of interstudy heterogeneity for OTUs. In the Asian population, GC tissues had fewer OTUs (*MD* = −69.62, 95%CI: −132.49 to −6.75) compared with non-cancer tissues, which was not observed in the non-Asian population (*MD* = 6.50, 95%CI: −13.61 to 26.61).

Sources of samples, sample size and *H. pylori* infection status contributed to heterogeneity for Chao 1. In the surgical subgroup, GC tissues had higher Chao 1 (*MD* = 83.08, 95%CI: 47.41 to 118.75), but no significant difference was observed in the endoscopic subgroup (*MD* = −282.36, 95%CI: −605.53 to 40.82). Lower Chao 1 (*MD* = −224.11, 95%CI: −419.91 to −28.31) in GC tissues was observed in the large sample size subgroup, but not in the small sample size subgroup (*MD* = 21.08, 95%CI: −49.74 to 91.89). In the *H. pylori* negative subgroup, Chao 1 (*MD* = −43.32, 95%CI: −56.92 to −27.72) significantly declined in GC tissues, while no significant difference was observed in the *H. pylori* positive subgroup (*MD* = 10.74, 95%CI: −22.67 to 44.16).

Sources of samples was also the major source of heterogeneity for the relative abundance of *Bacteroidetes* and *Fusobacteria*. In the surgical subgroup, *Bacteroidetes* (*MD* = 7.40, 95%CI: −2.39 to 12.41) and *Fusobacteria* (*MD* = 2.00, 95%CI: 0.98 to 3.02) was found to be enriched in GC tissues, while the differences in the abundance of *Bacteroidetes* (*MD* = 0.34, 95%CI: −4.13 to 4.81) and *Fusobacteria* (*MD* = 0.38, 95%CI: −0.61 to 1.37) was not significant in the endoscopic subgroup.

## Discussion

Consecutive alternations in the diversity and composition of gastric microbiota have been observed during GC development (50). In this meta-analysis, we identified the changes in the diversity and composition of gastric microbiome during gastric carcinogenesis based on 14 case-control studies with a total of 1,543 patients. The results demonstrated that alpha diversity declined during gastric carcinogenesis. Compared with non-cancer group, GC group had a higher abundance of *Firmicutes* at the phylum level, and enrichments of *Streptococcus* and *Lactobacillus* at the genus level. The relative abundance of the rest bacterial phyla or genera analyzed in our study did not significantly differ between groups.

Decline in the microbial diversity has been reported in a range of gastrointestinal diseases including inflammatory bowel disease (51, 52) and colorectal cancer (53), which could be a sign of microbial dysbiosis. We evaluted the the differences in four alpha diversity indexes between cancer and non-cancer groups. OTUs and Simpson index significantly declined in cancer group, and we also observed a non-significant downward trend in Chao 1. During gastric carcinogenesis, the disruption of gastric homeostasis leads to decreased gastric acidity and dysregulated metabolic functions (44, 54), which makes the microhabitats of the tumor site no longer suitable for the colonization of bacteria.

Previous studies have reported controversial results on differences of gastric microbiota composition between GC and non-cancer patients (9). Discrepancies across studies were comprehensively and quantitavely analyzed in the present meta-analysis. *Proteobacteria* and *Firmicutes* are two dominant bacterial phylum of the gastric microbiota in patients with or without GC (28, 38, 49). *Helicobacter* is the major component of *Proteobacteria*, and its abundance was found to be inversely correlated with the abundance of *Firmicutes* (47). The current view posits that gastric carcinogenesis is accompanied by a gradual loss of *Helicobacter* (especially *H. pylori*) colonization (10), which may explain the depletion of *Proteobacteria* and the enrichment of *Firmicutes* in the GC group as demonstrated by our analyses.

At the genus level, the GC tissue was characterized by significant enrichments of *Streptococcus* and *Lactobacillus*. *Streptococcus* is the first inhabitant of the human oral cavity (55). The abundance of *Veillonella*, which is another dominant genus of the oral microbiota (56), was higher in the cancer group, although it did not achieve statistical significance. Overabundance of oral bacteria was found to be correlated with a spectrum of malignancies including but not limited to colorectal cancer (57), pancreatic cancer (58, 59) and lung cancer (60). Though the relationship between oral microbiome and GC has not yet been clarified, oral microbiome has been considered as a potential biomarker for non-invasive diagnosis of GC (44). *Streptococcus* is also categorized as lactic acid bacteria (LAB) together with *Lactobacillus*. The enrichments of LAB increase microbially-derived lactate, which is not only an important energy source for cancer cells, but is also involved in multiple steps of carcinogenesis by promoting inflammation, angiogenesis, metastasis and immune evasion (61). Additionally, LAB may result in DNA damage by increasing the level of reactive oxygen species (ROS) (62).

Due to the diverse and dynamic nature of gastric microbiota, we conducted several subgroup analyses as well as meta-regression analyses to investigate potential sources of heterogeneity. Our analyses indicated that the source of samples (surgical vs. endoscopic) was the major source of heterogeneity for both alpha diversity indexes and abundance of bacterial phyla. Notably, peritumor non-cancer tissues in the surgical subgroup had even lower Shannon index, Chao 1 and fewer OTUs compared with tumor tissues, which is contrary to the overall findings as well as the results of the endoscopic subgroup. Considering that in studies based on surgical samples, the comparison of diversity was carried out between cancer tissues and peritumor non-cancer tissues, the above findings might be explained by the overgrowth of certain bacteria (for example, oral bacteria or LAB as identified by our analyses) at the tumor site in the context of gastric bacterial dysbiosis.

Our systematic review and meta-analysis quantitavely assesses the alterationsin the diversity and composition of gastric microbiota during gastric carcinogenesis. Hence, the present study has several clinical implications. Firstly, to clarify the changing regularity of gastric microbiota composition during carcinogenesis, and to identify specific microorganisms involved in this process, which may provide hints for the pathogenesis of GC and the exploration of potential microbial therapy targets. Secondly, the detection of changes in gastric microbiota, especially the overgrowth of certain bacteria (eg, *Streptococcus, Lactobacillus, Veillonella*), might assist the diagnosis of GC. The present study has several limitations that should be noted. Despite our best efforts, several studies were not included in the quantitative analysis due to lack of sufficient data, which might lead to bias to our results. Also, there is substantial heterogeneity across studies, even though the source of heterogeneity was partly identified by subgroup analyses. Moreover, gastric microbiota, especially non-*H. pylori* bacteria is a relatively young field, thus the number of studies and available data included are limited, adding to the difficulty to perform stable meta-analyses, subgroup analyses and meta-regression analyses, as well as to evaluate the correlation between gastric microbiota and the prognosis of GC which is an important but open clinical issue. Finally, we only enrolled observational studies, limiting the establishment of a cause-and-effect relationship.Therefore, with continuous publication of articles, the update of the meta-analysis is warranted.

## Conclusion

In summary, our review found that the diversity and composition of gastric microbiota differed between GC and non-GC tissues. Dysbiosis of gastric microbiota occurred in GC tissues, this was reflected by decreased alpha diversity and enrichments or depletions of certain bacteria. The update of meta-analysis is warranted with more articles published.

## Data Availability Statement

The original contributions presented in the study are included in the article/Supplementary Material, further inquiries can be directed to the corresponding authors.

## Author Contributions

YY: study concept and design, data extraction and interpretation, and drafting the article and critical revision. RJ: data extraction and statistical analysis and interpretation of the data and drafting the article. XZ: study design, statistical analysis, and revision of the article for important intellectual content. XC: data extraction and statistical analysis. QW and WZ: study design, and revision of the article for important intellectual content. QJ: revision of the article for important intellectual content. YZ: data extraction and interpretation of the data. XW and AY: study concept, design, and interpretation of the data revision of the article for important intellectual content. All the authors contributed to the review and revision of the manuscript and approved the submission.

## Funding

This work was supported by the National Natural Science Foundation of China (General Program, Grant Number 82073184), Peking Union Medical College Hospital Youth Program (Grant Number pumch201911356) and Beijing Science and Technology Program (Grant Number Z181100001618013). The sponsors have not been involved in study design, data collection, data analysis and result interpretation.

## Conflict of Interest

The authors declare that the research was conducted in the absence of any commercial or financial relationships that could be construed as a potential conflict of interest.

## Publisher's Note

All claims expressed in this article are solely those of the authors and do not necessarily represent those of their affiliated organizations, or those of the publisher, the editors and the reviewers. Any product that may be evaluated in this article, or claim that may be made by its manufacturer, is not guaranteed or endorsed by the publisher.
