# Supplementary material for: Alterations in Gastric Mucosal Microbiota in Gastric Carcinogenesis: A Systematic Review and Meta-Analysis
Source: Front Med (Lausanne). 2021 Dec 3;8:754959. doi: 10.3389/fmed.2021.754959 (PMC8678046; doi:10.3389/fmed.2021.754959)
Supplement: Supplementary file 2 [file Table_2.DOC]

**NEWCASTLE - OTTAWA QUALITY ASSESSMENT SCALE**

**CASE CONTROL STUDIES**

Note: A study can be awarded a maximum of one star for each numbered item within the Selection and Exposure categories. A maximum of two stars can be given for Comparability.

**Selection**

1) Is the case definition adequate?

a) yes, with both clinical and histological evaluations **🟑**

b) yes, eg record linkage or based on self-reports

c) no description

2) Representativeness of the cases

a) consecutive or obviously representative series of cases **🟑**

b) potential for selection biases or not stated

3) Selection of controls

a) community controls **🟑**

b) hospital controls

c) no description

4) Definition of controls

a) yes, with subdivision into normal mucosa, non-atrophic gastritis, atrophic gastritis, intestinal metaplasia and intraepithelial neoplasia **🟑**

b) yes, without further subdivision

c) no description

5) Does the study have adequate exclusion criteria

a) yes, have clear exclusion criteria, like history of surgery, history of taking antibiotics, prebiotics, probiotics, proton pump inhibitors (PPIs), chemotherapeutic drugs and any other drugs affecting gastric microbiota within the last month **🟑**

b) no description

6) Study size

a) ≥50 participants in each group **🟑**

b) <50 participants in each group

**Comparability**

1) Comparability of cases and controls on the basis of the design or analysis

a) study controls for *H.pylori* infection status **🟑**

b) study controls for age, sex, country or region, race/ethnicity**🟑**

**Exposure**

1) Ascertainment of the method

a) detailed description of experimental procedures**🟑**

b) description of quality control**🟑**

c) no description

2) Same method of ascertainment for cases and controls

a) yes **🟑**

b) no

3) Non-response rate

a) same rate for both groups **🟑**

b) non respondents described

c) rate different and no designation

**NEWCASTLE - OTTAWA QUALITY ASSESSMENT SCALE**

**COHORT STUDIES**

Note: A study can be awarded a maximum of one star for each numbered item within the Selection and Outcome categories. A maximum of two stars can be given for Comparability

**Selection**

1) Representativeness of the exposed cohort

a) truly representative of the gastric cancer population**🟑**

b) somewhat representative of the gastric cancer population **🟑**

c) selected group of users (eg, nurses, volunteers)

d) no description

2) Selection of the non-exposed cohort

a) drawn from the same community as the exposed cohort, with subdivision into normal mucosa, non-atrophic gastritis, atrophic gastritis, intestinal metaplasia and intraepithelial neoplasia**🟑**

b) drawn from the same community, without further subdivision

c) drawn from a different source

d) no description

3) Ascertainment of the method

a) detailed description of experimental procedures**🟑**

b) description of quality control**🟑**

c) no description

4) Demonstration that outcome of interest was not present at start of study

a) yes **🟑**

b) no

5) Does the study have adequate exclusion criteria

a) yes, have clear exclusion criteria, like history of surgery, history of taking antibiotics, prebiotics, probiotics, proton pump inhibitors (PPIs), chemotherapeutic drugs and any other drugs affecting gastric microbiota within the last month **🟑**

b) no description

6) Study size

a) ≥50 participants in each group **🟑**

b) <50 participants in each group

**Comparability**

1) Comparability of cohorts on the basis of the design or analysis

a) study controls for *H.pylori* infection status **🟑**

b) study controls for age, sex, country or region, race/ethnicity**🟑**

**Outcome**

1) Study design

a) prospective **🟑**

b) retrospective

2) Assessment of outcome

a) independent blind assessment **🟑**

b) record linkage **🟑**

c) self-report

d) no description

3) Adequacy of follow up of cohorts

a) complete follow up - all subjects accounted for **🟑**

b) subjects lost to follow up unlikely to introduce bias - small number lost - ≥90 % (select an adequate %) follow up, or description provided of those lost) **🟑**

c) follow up rate < 90% (select an adequate %) and no description of those lost

d) no statement
