# Supplementary material for: Alterations in Gastric Mucosal Microbiota in Gastric Carcinogenesis: A Systematic Review and Meta-Analysis
Source: Front Med (Lausanne). 2021 Dec 3;8:754959. doi: 10.3389/fmed.2021.754959 (PMC8678046; doi:10.3389/fmed.2021.754959)
Supplement: Supplementary file 3 [file Table_3.DOCX]

Supplementary Appendix3 Changes in relative abundance of bacterial genus

| **Bacterial genus** | **Studies (n)** | **^#^MD [95%CI]** | **P value** | **I^2^ (%)** |
| --- | --- | --- | --- | --- |
| *Helicobacter* | 7 | -13.40 [-28.24, 1.45] | 0.08 | 89 |
| *Streptococcus* | 5 | 3.03 [0.07, 6.00] | 0.04 | 66 |
| *Lactobacillus* | 5 | 5.15 [1.27, 9.04] | 0.009 | 40 |
| *Veillonella* | 4 | 0.76 [-0.39, 1.92] | 0.20 | 35 |
| *Prevotella* | 5 | -0.47 [-0.97, 0.03] | 0.07 | 43 |
| *Sphingomonas* | 5 | 0.01 [-0.20, 0.21] | 0.95 | 81 |
| *Fusobacterium* | 5 | 0.61 [-0.40, 1.61] | 0.24 | 82 |
| *Neisseria* | 4 | -0.13 [-0.44, 0.19] | 0.43 | 0 |

# A positive MD represents a higher relative abundance in gastric cancer group
