# Supplementary material for: Alterations in Gastric Mucosal Microbiota in Gastric Carcinogenesis: A Systematic Review and Meta-Analysis
Source: Front Med (Lausanne). 2021 Dec 3;8:754959. doi: 10.3389/fmed.2021.754959 (PMC8678046; doi:10.3389/fmed.2021.754959)
Supplement: Supplementary file 4 [file Table_4.docx]

**Supplementary Appendix 4 Subgroup analyses and univariate meta-regression analyses of changes in relative abundance of bacterial genera**

| **Group** | **Subgroups** | **Studies (n)** | **^#^MD [95% CI]** | **I^2^ (%)** | **^&^I^2^_sub_ (%)** | **^*^P** |
| --- | --- | --- | --- | --- | --- | --- |
| **Relative abundance of *Helicobacter*** | | | | | | |
| Age | Mean age <55 years | 5 | -10.61 [-24.75, 3.52] | 84 | 0 | 0.75 |
|  | Mean age ≥55 years | 2 | -18.14 [-62.27, 25.99] | 92 |  |  |
| Sources of samples | Endoscopic biopsies | 6 | -8.22 [-20.49, 4.05] | 81 | 91 | <0.001 |
|  | Surgical biopsies | 1 | -40.00 [-54.12, -25.88] | - |  |  |
| Sample size | Sample size <100 | 3 | -13.02 [-34.47, 8.42] | 67 | 0 | 0.96 |
|  | Sample size ≥100 | 4 | -13.70 [-34.01, 6.62] | 94 |  |  |
| Study quality | <8 scores by NOS | 4 | -17.03 [-40.14, 6.08] | 87 | 0 | 0.59 |
|  | ≥8 scores by NOS | 3 | -8.63 [-28.09, 10.83] | 87 |  |  |
| **Relative abundance of *Streptococcus*** | | | | | | |
| Age | Mean age <55 years | 3 | 3.22 [-2.59, 9.03] | 82 | 0 | 0.72 |
|  | Mean age ≥55 years | 2 | 2.11 [0.63, 3.58] | 0 |  |  |
| Sources of samples | Endoscopic biopsies | 4 | 3.47 [-1.15, 8.08] | 73 | 0 | 0.55 |
|  | Surgical biopsies | 1 | 2.00 [0.49, 3.51] | - |  |  |
| Sample size | Sample size <100 | 3 | 1.17 [-1.82, 4.17] | 10 | 11 | 0.29 |
|  | Sample size ≥100 | 2 | 5.12 [-1.56, 11.81] | 89 |  |  |
| Study quality | <8 scores by NOS | 4 | 4.02 [0.71, 7.33] | 67 | 72 | 0.06 |
|  | ≥8 scores by NOS | 1 | -1.37 [-5.91, 3.17] | - |  |  |
| **Relative abundance of *Lactobacillus*** | | | | | | |
| Age | Mean age <55 years | 4 | 5.76 [0.74, 10.79] | 53 | 0 | 0.90 |
|  | Mean age ≥55 years | 1 | 5.16 [-3.07, 13.39] | - |  |  |
| Sample size | Sample size <100 | 3 | 8.39 [2.30, 14.48] | 0 | 42 | 0.19 |
|  | Sample size ≥100 | 2 | 3.42 [-0.85, 7.68] | 51 |  |  |
| Study quality | <8 scores by NOS | 3 | 4.54 [-0.80, 9.88] | 47 | 0 | 0.48 |
|  | ≥8 scores by NOS | 2 | 7.35 [1.73, 12.96] | 0 |  |  |
| **Relative abundance of *Veillonella*** | | | | | | |
| Age | Mean age <55 years | 3 | 1.22 [-0.60, 3.04] | 33 | 9 | 0.30 |
|  | Mean age ≥55 years | 1 | -0.04 [-1.54, 1.46] | - |  |  |
| Sample size | Sample size <100 | 3 | 0.75 [-1.65, 3.15] | 46 | 0 | 0.82 |
|  | Sample size ≥100 | 1 | 1.05 [0.24, 1.86] | - |  |  |
| Study quality | <8 scores by NOS | 3 | 0.76 [0.06, 1.46] | 0 | 63 | 0.10 |
|  | ≥8 scores by NOS | 1 | 4.94 [-0.01, 9.89] | - |  |  |
| **Relative abundance of *Prevotella*** | | | | | | |
| Age | Mean age <55 years | 4 | -0.57 [-1.59, 0.45] | 56 | 0 | 0.70 |
|  | Mean age ≥55 years | 1 | -0.35 [-0.77, 0.07] | - |  |  |
| Sample size | Sample size <100 | 3 | -0.03 [-2.38, 2.32] | 17 | 0 | 0.64 |
|  | Sample size ≥100 | 2 | -0.64 [-1.63, 0.36] | 77 |  |  |
| Study quality | <8 scores by NOS | 3 | -0.28 [-0.61, 0.04] | 0 | 0 | 0.71 |
|  | ≥8 scores by NOS | 2 | 0.78 [-4.87, 6.43] | 63 |  |  |
| **Relative abundance of *Sphingomonas*** | | | | | | |
| Age | Mean age <55 years | 3 | -2.02 [-5.61, 1.57]  0.67 [0.08, 1.26] | 83 | 34 | 0.22 |
|  | Mean age ≥55 years | 2 | 0.27 [-0.37, 0.91] | 80 |  |  |
| Sample size | Sample size <100 | 2 | -1.99 [-5.62, 1.64] | 84 | 28 | 0.24 |
|  | Sample size ≥100 | 2 | 0.23 [-0.52, 0.97] | 85 |  |  |
| Study quality | <8 scores by NOS | 4 | -0.01 [-0.15, 0.12] | 67 | 92 | <0.001 |
|  | ≥8 scores by NOS | 1 | -8.43 [-13.18, -3.68] | - |  |  |
| **Relative abundance of *Fusobacterium*** | | | | | | |
| Age | Mean age <55 years | 3 | 1.35 [-1.36, 4.07] | 71 | 0 | 0.70 |
|  | Mean age ≥55 years | 2 | 0.79 [-0.09, 1.67] | 25 |  |  |
| Sources of samples | Endoscopic biopsies | 4 | 0.53 [-0.94, 2.01] | 56 | 0 | 0.56 |
|  | Surgical biopsies | 1 | 1.00 [0.47, 1.53] | - |  |  |
| Sample size | Sample size <100 | 3 | 1.51 [-1.26, 4.28] | 57 | 0 | 0.46 |
|  | Sample size ≥100 | 2 | 0.38 [-0.77, 1.54] | 94 |  |  |
| Study quality | <8 scores by NOS | 4 | 0.38 [-0.66, 1.41] | 84 | 49 | 0.16 |
|  | ≥8 scores by NOS | 1 | 2.27 [-0.16, 4.70] | - |  |  |
| **Relative abundance of *Neisseria*** | | | | | | |
| Age | Mean age <55 years | 3 | -0.12 [-0.44, 0.20] | 0 | 0 | 0.77 |
|  | Mean age ≥55 years | 1 | -0.58 [-3.61, 2.45] | - |  |  |
| Sample size | Sample size <100 | 3 | 3.01 [-2.53, 8.56] | 91 | 0 | 0.51 |
|  | Sample size ≥100 | 1 | 1.05 [-0.72, 2.82] | 0 |  |  |
| Study quality | <8 scores by NOS | 3 | 0.26 [-0.88, 1.40] | 0 | 0 | 0.49 |
|  | ≥8 scores by NOS | 1 | -0.16 [-0.49, 0.17] | - |  |  |

# A positive MD represents a higher relative abundance in gastric cancer group

& Heterogeneity across subgroups

* P value of univariate meta-regression analyses which test for subgroup differences
